# Health facilities at the district level in Indonesia

**DOI:** 10.1186/1743-8462-6-13

**Published:** 2009-05-18

**Authors:** Peter Heywood, Nida P Harahap

**Affiliations:** 1 Menzies Centre for Health Policy, University of Sydney, NSW, Australia; 2 Jalan Bukit Dago Selatan, Bandung. West Java Province, Indonesia

## Abstract

**Background:**

At Independence the Government of Indonesia inherited a weak and unevenly distributed health system to which much of the population had only limited access. In response, the government decided to increase the number of facilities and to locate them closer to the people. To staff these health facilities the government introduced obligatory government service for all new graduates in medicine, nursing and midwifery. Most of these staff also established private practices in the areas in which they were located. The health information system contains little information on the health care facilities established for private practice by these staff. This article reports on the results of enumerating all health facilities in 15 districts in Java.

**Methods:**

We enumerated all healthcare facilities, public and private, by type in each of 15 districts in Java.

**Results:**

The enumeration showed a much higher number of healthcare facilities in each district than is shown in most reports and in the health information system which concentrates on public, multi-provider facilities. Across the 15 districts: 86% of facilities were solo-provider facilities for outpatient services; 13% were multi-provider facilities for outpatient services; and 1% were multi-provider facilities offering both outpatient and inpatient services.

**Conclusion:**

The relatively good distribution of health facilities in Indonesia was achieved through establishing public health centers at the sub-district level and staffing them through a system of compulsory service for doctors, nurses and midwives. Subsequently, these public sector staff also established solo-provider facilities for their own private practice; these solo-provider facilities, of which those for nurses are almost half, comprise the largest category of outpatient care facilities, most are not included in official statistics. Now that Indonesia no longer has mandatory service for newly graduated doctors, nurses and midwives, it will have difficulty maintaining the distribution of facilities and providers established through the 1980s. The current challenge is to envision a new health system that responds to the changing disease patterns as well as the changes in distribution of health facilities.

## Background

At Independence the Government of Indonesia inherited a weak and unevenly distributed health system to which much of the population had only limited access. In response, the government decided to increase the number of facilities and to locate them closer to the people. In doing so they were recognizing that access to facilities was limited for a combination of geographic and economic reasons – the incidence of poverty was high [1] and public transport infrastructure [2] very limited and inadequate. Further, the ideological climate of the time, reflecting experience under the Dutch colonial system, distrusted market forces as the mechanism for deciding the allocation of resources among competing uses; there was a complementary belief that civil servants could make such decisions [3]. These two forces combined to produce a health system plan with a heavy emphasis on public funding and provision.

In the early 1950s health facilities consisted mainly of: hospitals (both public and private); treatment clinics (balai pengobatan), most were government owned and concentrated on treatment of adults; and maternal and infant health clinics (balai kesehatan ibu dan anak), also mostly government owned; the orientation of the system was very heavily curative. In 1951 a new health program for the city of Bandung was introduced – the principle was integration of preventive and curative medicine [4]. This new health system, eventually widely known as the Bandung Plan, became the blueprint for a new national health system. Based on the Bandung Plan, the strategy that emerged was to establish a network of public health facilities throughout the country with a health center at the sub-district level and a hospital at the district level. Initially this involved building on the institutions that already existed – the central, and eventually iconic, institution, the health center, was initially created through the amalgamation of existing general treatment clinics (balai pengobatan – BP) and maternal and child health clinics (balai kesehatan ibu dan anak – BKIA). To the activities of this combined unit were added preventive activities which were more in the nature of public goods. Subsequently, new health centers in which to house these activities were built. The goal was to establish and staff a health center in each of the more than 7000 sub-districts of the country. Implementation of the Bandung Plan from the mid-1950s meant that in 1969, at the start of the First Five Year Plan, Indonesia already had 1058 health centers, 7590 treatment clinics (balai pengobatan) and 5620 maternal and infant health clinics [5]. In this way Indonesia rapidly established a network of health centers at the sub-district level and hospitals at the district level.

Through successive 5 Year Plans the number of health centers increased as a result of both mergers, where there were treatment and maternal and infant health clinics in the same locality, and through the building and staffing of new facilities where there had been none previously. By the mid-1990s there were more than 7000 health centers. The average population per health center had fallen from 96,000 in 1968 to under 30,000 in 1995 [6]. In addition, the government added over 20,000 health sub-centers and during the 1980s started a program to locate midwives in villages, a program in which the village midwife is totally synonymous with a new type of facility. Construction of this network of public facilities involved a substantial investment which was made using budget allocations under Presidential Instruction (Inpres).

As this new network of public health facilities was established the task was to staff them. Not only was there a modest increase in the total number of facilities but the staffing complement for each facility changed markedly. Now it was envisaged that each health center would be lead by a doctor and that there would be a standard complement of nurses and midwives as well as other paramedical and administrative staff. Critically, there was a marked increase in the number of doctors, nurses and midwives needed to carry out the clinical and preventive functions and they would have to be distributed to more remote areas of the country outside Java/Bali and Sumatra. To achieve the required numbers and distribution of staff the government, in 1974, introduced obligatory government service for all new graduates in medicine, nursing and midwifery [7,8]. The new graduates were made permanent civil servants and assigned to work in these various health facilities for the first 3 years of their service if on Java, for lesser periods in areas outside Java – see [9] for further details of human resources at the district level in Indonesia.

In this way the government achieved a much better distribution of health facilities and, through the compulsory service, the staff needed to deliver services. In addition to these public facilities there were also private hospitals and private treatment clinics. Referral hospitals were either present at the provincial and national levels or new ones were established. Over time state owned enterprises, the armed forces and police also established their own health systems, with a heavy emphasis on hospitals.

It is these public, and a limited number of private, facilities (especially hospitals) that are usually counted when there is discussion of health facilities. However, this is only part of the story. An equally important part of the story relates to the health facilities established by the providers allocated to staff these public institutions, hospitals and health centers, especially the latter. These staff were not highly paid and, to supplement their income, the government allowed private practice after official working hours for doctors and midwives. Thus, the private practices of these public servants became an important source of healthcare facilities for ambulatory care. Despite their apparent importance these facilities are seldom referred to when healthcare facilities are discussed in Indonesia.

The private treatment clinics operated by the private sector before the new health system was created also continued to function and, in some areas, new private treatment clinics have been set up. Other private sector facilities that still operate, but are seldom discussed, include private maternity hospitals and clinics. Finally, there is now a growing group of doctors and midwives who are not employed by the government and have established health facilities to house their private practices.

The health information system in Indonesia has always been heavily oriented to the public sector. Moreover, with the radical decentralization of government functions following the fall of the authoritarian regime of President Suharto [10], and the autonomy of district governments that followed, information about the public system is now more limited and of uneven reliability, there is only limited information about the private sector, and understanding of how the health system overall is developing is less complete than previously.

The work reported here is part of an attempt to understand what is happening at the district level in the health sector since decentralization starting with a basic enumeration of the human resources and the health facilities in which they work and deliver services. Our aim, in a sample of 15 districts in Java, is to: (1) enumerate the stock of health facilities (public and private) in the health sector in 2006; (2) enumerate the stock of human resources (public and private) in the health sector in 2006; and (3) estimate the funds (public and private) spent on health care in the course of 2006. This article reports on the results of enumerating health facilities in 15 districts in Java. Separate articles report on health personnel [9] and funding at the district level [11].

## Methods

As much of the information we wished to collect is not available from the central government we collected it in the districts. This work concentrates on Java where 60% of the population lives. Resources were sufficient to allow data to be collected in 15 districts. To ensure representation of the range of situations in Java 5 districts were chosen in each of West Java Province, Central Java Province and East Java Province. Basic details of the 15 districts are shown in Table 1.

**Table 1:** Basic information about the 15 districts included in this study.

Province	District	Population	No. Sub-districts
West Java	Ciamis	1458680	36
	Cirebon	2134656	37
	Garut	2274973	41
	Subang	1402134	22
	Sukabumi	2240901	45
Central Java	Brebes	1727708	17
	Cilacap	1717273	24
	Jepara	1078037	14
	Pemalang	1341422	14
	Rembang	591786	14
East Java	Jombang	1203716	21
	Ngawi	857449	19
	Pamekasan	782917	13
	Sampang	801541	14
	Trenggalek	682328	14

The 15 districts were selected as follows. Between 1997 and 2004 East Java Province and Central Java Province were included in a World Bank Safe Motherhood Project [12]. The endline data for this project were collected in 5 districts in each province (a total of 10 districts) at the time of the 2002–03 Demographic and Health Survey^1 ^(DHS) [13]. The districts for the endline data collection were selected purposively by the Safe Motherhood Project team to illustrate the range of settings in which the project was implemented. The sample size in these districts was sufficient to provide district level estimates of the basic indicators in the DHS. The DHS was repeated in the same districts, with oversampling, in 2007. (A comparison of 2002–03 and 2007 DHS results for these 10 districts will be presented in a separate article. [Heywood, P. Changes in health system performance in 10 districts in Java, Indonesia, unpublished.]). West Java was not included in the earlier Safe Motherhood Project. However, in 2007 oversampling for the DHS was also carried out in 5 West Java districts. The districts were selected purposively to illustrate the range of district settings in West Java. Table 1 shows the province, population and number of sub-districts in each of the 15 districts included in this study. Using the World Bank classification [14] all 15 districts have low fiscal revenue per capita; Cilacap and Subang have high Gross District Product per capita, the other 13 districts all have low Gross Development Product per capita.

Data were collected by three teams – one for each province. The provincial team leaders were from, and based in, the province, and had previous experience in collecting health data at the district level.

The goal was to enumerate in each of the 15 districts all health facilities^2^, as defined in Table 2. This included two basic types of facility: first, multiple-provider facilities, that is facilities with more than one healthcare professional; this information was obtained from the District Health Profile prepared annually by the district health office. Second, solo-provider facilities, that is, facilities through which healthcare professionals (doctors, nurses and midwives) working in the district operate their solo private^3 ^practices. These solo-provider facilities are of two types – the private practice of the staff of public facilities, and the private practices of those who work primarily on their own account. Thus, there is a range of health facility types in each district. Enumerating solo-provider facilities involved identifying district health personnel (public and private) and determining whether or not they engage in private practice.

**Table 2:** Definitions of health facilities by type.

Health facility	Description	Public/Private
A. Multiple-provider facilities		
Public hospital [Rumah Sakit Umum Daerah (RSUD)]	Public hospital located at the district level	Public
Private hospital [Rumah Sakit Swasta (RSUS)]	Private hospital located at the district level, national and provincial government enterprises, police, defence forces.	Private
Hospital for women and children [Rumah Sakit Ibu dan Anak (RSIA)]	Private hospital for women and children located in the district.	Private
Women's hospital [Rumah Sakit Bersalin (RSB)]	Private women's hospital located in the district.	Private
Maternity clinic [Rumah Bersalin (RB)]	Private maternity clinics with more than 2 beds.	Private
Health center [Pusat Kesehatan Masyarakat (Puskesmas)]	Public health center located in the district – in general they are located at the sud-district level.	Public
Auxiliary health center [Puskesmas pembantu (Pustu)]	Public auxiliary health center – in general they are located at the sub-district level, usually in a village.	Public
Treatment clinic [Balai pengobatan (BP)]	Treatment clinic. Before the advent of the puskesmas there were private and public treatment clinics. As the puskesmas was developed the public treatment clinics were incorporated in the puskesmas with the result that only the private balai pengobatan remained. Although they have been ignored by the government and donors they remain a significant source of treatment, especially in urban areas. They are licensed by the local government and must have a doctor as the supervisor. In practice, most of the doctors named as the supervisor seldom visit and nurses, and some midwives, provide most of the health care unsupervised.	Private
		
B. Solo-provider facilities		
Village midwife [Bidan di desa (BDD)/Pondok Bersalin Desa (Polindes)]	BDD is a village midwife who receives a government salary and also may charge for the services she provides and retain the fee herself. Although the village midwife theoretically lives in the village (desa) there are reports indicating that in many villages she lives elsewhere, maybe in a nearby urban area. The services provided by the BDD may be offered in a room in her house or in a structure in that is the property of, and was built by, the village government (polindes). In the polindes the services are provided by the village midwife who charges for the services and retains the fees.	Private
Doctor in full-time private practice.	Doctor whose primary professional activity is private practice and who does not receive a salary from the government.	
Doctor in part-time private practice.	Doctor whose primary professional appointment is with the government to work in a government health facility and who also has a part-time private practice after office hours.	Private
Nurse in part-time private practice	Nurse whose primary professional activity is in a public or private health facility and who has a part-time private practice after hours.	Private
Midwife in full-time private practice	Midwife whose primary professional activity is private practice and who does not receive a salary from the government.	Private

Health facility is defined as a physical structure (which varies from a large complex of buildings to a single room in a house from which health services are offered by a doctor, nurse or midwife).

The primary source of data on district health personnel was the district health office and the district hospital. There are two basic documents usually available at each district health office and district hospital – a list of all government employees in the sector by rank and seniority (*Daftar Nominatif*), and the list of all permanent civil servants in the district by sector (*Daftar Urut Kepangkatan*, also known as the *DUK*). All healthcare providers who do not work for the government but have a private practice in which healthcare is provided should be licensed by the district government and our list was supplemented from those sources as well. Whilst these lists were kept more or less up to date in the past, since decentralization many districts put much less effort into these tasks. Consequently there is considerable variation between districts (and provinces) in the completeness of these lists today. In some districts where the government records were clearly incomplete we also consulted the membership lists from the professional associations for doctors, nurses and midwives – these lists potentially include members in both the public (because public sector doctors, nurses and midwives are members of the associations) and private (because doctors and midwives have private practice rights) sectors and are also in varying states of completeness. Regardless of the source of information, all names on the membership lists were checked against the public sector lists to minimize double counting and ensure that those on the list were still offering health care services. Thus, a consolidated list of doctors, nurses and midwives (see Table 3 for definitions) was produced for each district. For each provider we also recorded their employment status (civil servant, contract, volunteer, self employed) and primary place of work (hospital, health center, private practice, clinic). In West Java this information is essentially complete. In the other two provinces, East Java and Central Java, there were districts in which the information on each provider did not include employment status and/or primary place of work. The aggregate information on employment status and primary place of work for the districts in these provinces is based on information available in the annual district health sector report and discussions with senior administrators in the district health office. Full details of the health personnel in these 15 districts have been published separately [9].

**Table 3:** Definitions of health service providers.

Provider	Description
Doctor (Dokter)	Graduate of an Indonesian medical school licensed by the government.
Nurse (Perawat)	Graduate of: (i) a Sekolah Perawat Kesehatan (SPK), students enter at the end of junior high school and the SPK training is regarded as equivalent to senior high school; OR (ii) an Akademi Perawatan for which students enter at the end of senior high school; OR (iii) Fakultas Ilmu Keperawatan, a university level course at the first degree level, there is a small number of second degree level graduates as well. All these institutions must be licensed by the government.
Midwife (Bidan)	Graduate of: (i) Sekolah Bidan (SB), students enter at the end of junior high school and this training is regarded as equivalent to senior high school; OR (ii) Program Pendidikan Bidan (PPB) – entrants to this 1 year program have an SPK nursing qualification; OR (iii) Akademi Kebidanan (Akbid) for which students enter at the end of senior high school. Originally midwives were trained as SB until this program was closed in 1984. After a 5 year period of no training of midwives the government started training again in 1989 through the PPB as village midwives; the PPB was closed in 1998 and was replaced by the Akbid program.

Determining the number of facilities which house the private practice of a nurse presents a particular problem because private practice by nurses is illegal. Even so, nurses are widely acknowledged to engage in private practice after their regular hours at a multi-provider facility such as a hospital or health center [8]. Because this practice is illegal nurses are reluctant to admit to the practice and estimates based on their own report are certain to be gross underestimates. An anthropological study [15] in one district in Central Java found that 90% of nurses maintained active private practices. There is general agreement in the community and amongst district health officials that private practice by nurses is very common. Given this background and to ensure that the estimate we produce is conservative we assumed that the proportion of nurses engaged in private practice is 60%. Discussions with health officials at district and provincial level confirm that this estimate is likely to underestimate the number of nurses in private practice and, therefore, the number of solo-provider facilities from which nurses provide services.

Using this approach, some providers (doctor, nurse, midwife) can contribute to the results in two ways: first as staff of a multi-provider facility (for example, a hospital, health center); and second, as the provider in a solo-provider facility (for example, a doctor or nurse in part-time private practice). Other providers, such as doctors and midwives in full-time private practice, will only contribute once through the single facility at which they work.

## Results

The results are summarized across the 15 district in Tables 4, 5, 6, and 7. (The results are shown in more detail by district and type of facility for the three provinces separately in Tables 8, 9, and 10.)

**Table 4:** Health facilities by type and public/private – across 15 districts

Facility type	Public (number)	Private (number)	Total (number)	Percent
Multiple-provider facilities for both inpatients and outpatients	19	111	130	1
Multiple-provider facilities for outpatients only	1334	458	1792	13
Solo-provider facilities for outpatients only	0	11577	11577	86
Total	1353	12146	13499	100
Percent	10	90	100	

Source: Authors' calculations.

Note: these facility types are listed in order of decreasing complexity of services offered.

**Table 5:** Solo-provider facilities for outpatients only – across 15 districts

Facility type	Number	Percent
Doctor in full-time private practice	847	7
Doctor in part-time private practice	1430	13
Village midwife [Bidan di desa (BDD)/Pondok Bersalin Desa (Polindes)]	3382	29
Nurse in part-time private practice	5325	46
Midwife in full-time private practice	593	5

Total	11577	100

Source: Authors' calculations.

Note: these facility types are listed in decreasing order of complexity of services offered.

**Table 6:** Multiple-provider facilities offering services for both inpatients and outpatients

	Number	Percent
Public hospital (Rumah Sakit Umum Daerah (RSUD))	19	15
Private hospital (Rumah Sakit Swasta (RSUS))	33	25
Hospital for women and children (Rumah Sakit Ibu dan Anak (RSIA))	2	2
Women's hospital (Rumah Sakit Bersalin (RSB))	8	6
Maternity clinic (Rumah Bersalin (RB))	68	52

Total	130	100

Source: Authors' calculations.

Note: these facility types are listed in decreasing order of complexity of services offered.

**Table 7:** Multiple-provider facilities for outpatients only – across 15 districts

	Number	Percent
Health center [Pusat Kesehatan Masyarakat (Puskesmas)]	504	28
Treatment clinic [Balai pengobatan (BP)]	458	26
Auxiliary health center [Puskesmas pembantu (Pustu)]	830	46

Total	1792	100

Authors' calculations.

Note: these facility types are listed in decreasing order of complexity of services offered.

**Table 8:** West Java.

Health facility	Public**/** Private	Ciamis	Cirebon	Garut	Subang	Sukabumi
A. Multiple-provider facilities.						
Public hospital (Rumah Sakit Umum Daerah (RSUD))	Public	1	2	1	1	3
Private hospital (Rumah Sakit Swasta (RSUS))	Private	3	4	1	2	2
Hospital for women and children (Rumah Sakit Ibu dan Anak (RSIA))	Private	0	0	0	0	0
Women's hospital (Rumah Sakit Bersalin (RSB))	Private	0	1	0	0	0
Maternity clinic (Rumah Bersalin (RB))	Private	2	5	2	3	11
Health center (Pusat Kesehatan Masyarakat (Puskesmas))	Public	51	53	62	39	57
Auxiliary health center (Puskesmas pembantu (Pustu))	Public	82	64	132	73	98
Treatment clinic (Balai pengobatan (BP))	Private	75	161	8	22	47
Sub-total (multiple-provider facility)		214	290	206	140	218
						
B. Solo-provider facilities						
Village midwife (Bidan di desa (BDD)/Pondok Bersalin Desa (Polindes))	Private	273	400	305	253	283
Doctor in full-time private	Private	38	188	53	95	99
Doctor in part-time private practice	Private	58	107	92	78	107
Nurse in part-time private practice	Private	501	480	590	451	353
Midwife in full-time private practice	Private	33	227	18	43	37
Sub-total (solo-provider facility)		903	1402	1058	920	879

Total		1117	1692	1264	1060	1097

Number of health facilities by type and district.

Source: Authors' calculations.

**Table 9:** Central Java.

Health facility	Public/Private	Brebes	Cilacap	Jepara	Pemalang	Rembang
A. Multiple-provider						
Public hospital (Rumah Sakit Umum Daerah (RSUD))	Public	1	2	1	1	1
Private hospital (Rumah Sakit Swasta (RSUS))	Private	5	3	2	2	0
Hospital for women and children (Rumah Sakit Ibu dan Anak (RSIA))	Private	0	0	1	0	0
Women's hospital (Rumah Sakit Bersalin (RSB))	Private	1	4	1	0	0
Maternity clinic (Rumah Bersalin (RB))	Private	7	6	3	12	4
Health center (Pusat Kesehatan Masyarakat (Puskesmas))	Public	28	36	20	22	16
Auxiliary health center (Puskesmas pembantu (Pustu))	Public	62	78	44	62	135
Treatment clinic (Balai pengobatan (BP))	Private	9	49	44	18	4
Sub-total (multiple-provider facility)		113	178	116	117	160
B. Solo-provider facilities						
Village midwife (Bidan di desa (BDD)/Pondok Bersalin Desa (Polindes))	Private	343	257	151	228	0
Doctor in fill-time private practice	Private	44	67	3	38	11
Doctor in part-time private practice	Private	137	116	127	92	81
Nurse in part-time private practice	Private	359	524	331	311	197
Midwife in full-time private practice	Private	35	0	0	0	9
Sub-total (solo-provider facility)		918	964	612	669	298

Total		1031	1142	728	786	458

Number of health facilities by type and district.

Source: Authors' calculations.

**Table 10:** East Java.

Health facility	Public/Private	Jombang	Ngawi	Pamekasan	Sampang	Trenggalek
A. Multiple-provider facilities.						
Public hospital (Rumah Sakit Umum Daerah (RSUD))	Public	1	1	1	1	1
Private hospital (Rumah Sakit Swasta (RSUS))	Private	6	1	0	0	2
Hospital for women and children (Rumah Sakit Ibu dan Anak (RSIA))	Private	0	0	1	0	0
Women's hospital (Rumah Sakit Bersalin (RSB))	Private	1	0	0	0	0
Maternity clinic (Rumah Bersalin (RB))	Private	0	9	4	0	0
Health center (Pusat Kesehatan Masyarakat (Puskesmas))	Public	34	24	20	20	22
Auxiliary health center (Puskesmas pembantu (Pustu))	Public	0	0	0	0	0
Treatment clinic (Balai pengobatan (BP))	Private	0	14	3	4	0
Sub-total (multiple- provider facility)		42	49	29	25	25
B. Solo-provider facilities						
Village midwife (Bidan di desa (BDD)/Pondok Bersalin Desa (Polindes))	Private	205	134	252	126	172
Doctor in fill-time private practice	Private	153	36	17	5	0
Doctor in part-time private practice	Private	148	96	70	48	73
Nurse in part-time private	Private	346	312	179	175	215
Midwife in full-time private practice	Private	77	22	87	5	0
Sub-total (solo-provider facility)		929	600	605	359	460

Total		971	649	634	384	485

Number of health facilities by type and district.

Source: Authors' calculations.

The summary across all 15 districts and individual facility types is shown in Table 4. Multiple-provider facilities for inpatient and outpatient services comprise less than 1% of all facilities and are both public and private; the most numerous are maternity hospitals and clinics (see Table 6), all of which are privately owned. Multiple-provider facilities offering outpatient services constitute 13% of the facilities; they are both public and private sector, but those in the public sector pre-dominate (Table 7). Facilities that house solo-providers of outpatient services comprise 86% of all the health facilities – they are all private.

There are 5 groups of solo-provider health facilities (Table 5). The largest single group, nurses, comprises almost half the total, 46%; the second largest group is village midwives at 29%. Private practice of doctors constitutes 19% (7% full time and 12% part time) of the solo-provider practices; and midwives in full-time practice are 5%.

The predominance of solo-providers of outpatient services means that most facilities are in the private sector; when taken together with the private sector facilities in other categories – multi-provider facilities offering both outpatients and inpatient services – 90% of all facilities are in the private sector (Table 4).

Multi-provider facilities which offer only outpatient services (Table 7) are dominated by the health center and associated auxiliary health center – 74% of this facility category; both are publicly funded. Privately operated treatment clinics are 26% of this category.

Of the facilities providing general inpatient and outpatient services (Table 6), hospitals are 40%, an average of 1 public hospital and 1–2 private hospitals per district. Sixty percent of this category are smaller facilities specifically for women and children – maternity clinics are the most common with an average of almost 4 per district.

There is considerable variation between districts in the proportion of the facilities contributed by each of these groups (see Tables 8, 9, and 10), depending in part on the degree of isolation and general income levels in the district.

## Discussion

Development of the publicly funded portion of the health system in post-Independence Indonesia was achieved essentially through the creation of a new facility, the health center, and then locating these facilities at the sub-district level throughout the country. At the same time a public hospital was established in districts in which there was no public hospital previously. These facilities were staffed with doctors, nurses and midwives through a period of obligatory service for all new graduates who were assigned to specific facilities. To supplement their incomes the government granted doctors and midwives rights to private practice; although nurses were not granted these rights, most of them established practices after hours as well and for the same reasons, their incomes were low too. The private practices of these public sector employees, together with an increasing number of private practitioners who do not work for the government, constitute the solo-provider facilities – in essence, the provider is the facility; these facilities, all of them private, account for 86% of facilities (Table 4). In addition, private hospitals and maternity clinics have also been established, often owned and staffed by public sector employees with the result that 90% of all facilities are private.

Thus, the common pattern of health facilities in most districts on Java includes a public hospital, one or two (usually smaller) private hospitals and three or four small private facilities offering inpatient and outpatient obstetric services to women and their young children. At the district level there are also private outpatient treatment clinics and numerous solo-provider private facilities for outpatient services; the providers are various, including doctors, nurses and midwives, some of whom are full-time private practitioners and others part-time in addition to their public sector roles.

At the sub district level there is a public health center, the associated auxiliary health centers, and a much larger number of solo-provider outpatient facilities through which doctors, nurses and midwives operate their after-hours private practices. Village midwives located in many villages also operate private practices.

This distribution of facilities and providers means that within each sub-district there is a range of facility types (multi-provider, solo-provider); a range of provider types in the facilities (doctors, nurse and midwives); a range of facility locations, some close to, even in the village, others in the sub-district headquarters. Some facilities are public, others private; some are free, at others there is a charge for the service. This distribution allows consumers to exercise some choice of facility and/or provider. In exercising this choice, many consumers choose the solo-provider facilities even though the public facilities at the health center, sub-center and district hospital are nominally free and they have to pay out-of-pocket at the private practice of the doctor, nurse and midwife. The lower fees of the nurse and midwife mean that they are often the preferred choice of the poor.

An extraordinary characteristic of the Indonesian system is that the most numerous of the solo-provider facilities – that staffed by nurses – is illegal and, therefore, seldom discussed. However, it is widely acknowledged that they do have private practice. Because it is not legal for them to provide treatment the government does not collect information about the solo-provider facilities of nurses and the nurses themselves are unwilling to acknowledge their own activities for fear of the law. In this study we assume that 60% of nurses have a private practice in which they offer treatment. Discussions with health authorities at the provincial and district levels indicate that this estimate may be conservative. Using this estimate, the solo-provider facilities of nurses are the largest single group of facilities (40% of all facilities and 46% of single provider facilities), much higher than village midwives who constitute a quarter of all facilities. Further, two-thirds of all nurses in a district are located at the sub-district level in the health center [9]; their private practice is most likely to be in the same locality meaning that these practices are quite widely distributed throughout the district. Despite constituting such a high proportion of all facilities, the system continues to act as if solo-provider facilities of nurses do not exist. If the past is any guide, enforcement of the law on private practice of nurses is unlikely to occur. So why not recognize reality and change the law? Attempts to do so have been vigorously opposed by both doctors and midwives in the past as they protect their vested interests and are likely to be opposed in the future, particularly if the government continues to take an ambivalent stance on the issue. It seems that the only option for nurses is to continue to operate outside the law until the government provides the leadership necessary to change the relevant legislation.

One category of facility about which very little is known is the private treatment clinic, usually found at the district level. They are usually staffed by nurses working outside their hours in public hospitals and health centers. These facilities operate within the law as the nurses are, on paper, under the supervision of a doctor. The supervision is usually very nominal and at a distance. Private treatment clinics are potentially an important source of outpatient care, particularly in urban areas. Indeed, across these 15 districts they are almost as numerous as the health centers. Little attention is paid to these facilities by the government in terms of either supervision or as a potentially innovative service delivery model.

There is one more element to the solo-provider facilities situation. That is the increasing number of solo-private providers who have no employment with the government and are usually located in the vicinity of the district capital. This is particularly true for doctors – in 6 of the districts studied more than one-third of the doctors are in private practice and do not receive a salary from the government; in 2 districts these private practitioners constitute more than half the doctors. This trend is also increasingly apparent for midwives (in 6 of the districts studied here more than 10% of the midwives were in private practice and did not receive a salary from the government; in 2 districts the proportion was as high as one-third) [9].

These private practitioners operate with minimal government supervision. Regulation and accreditation of facilities at which health services are provided is not well developed – to the extent that it occurs it concentrates on multiple-provider facilities. There is practically no supervision or accreditation of solo-provider facilities where the majority of outpatient services are provided.

The quality of services provided by all three professional groups is sub-optimal. Measuring quality in terms of knowledge about clinical guidelines, Barber et al showed low knowledge of evidence-based practices in all professional groups, particularly for prenatal and adult curative care [16]. Physicians had the highest scores. Nurses had lower scores than midwives and physicians. Whilst this work underscores that all three groups scored poorly, the most important point is that nurses, the largest single group of solo-provider facilities scored the lowest but attempts to improve their skills are opposed on the grounds that their private practice is illegal. Yet improving the quality of care provided by nurses in solo-provider facilities, where the low tariff makes them the facility of choice for many of the poor, may be one of the most important avenues for improving quality of outpatient care, especially for those with low incomes.

In terms of the contribution to seeing patients, each group-provider facility, because it houses a number of providers, has the capacity to see many more patients in a day than a solo-provider facility. Whether this is reflected in actual patients seen is not known as there is essentially no information on the characteristics of the solo-provider facilities, the number of patients they see in a day, the setting in which the services are provided, details of the actual services offered. What we do know is that 55% of ambulatory care is provided by private providers [14]. Further, informal observations and anecdotes indicate that many solo-provider practices regularly see more patients in a day than are routinely seen per provider per day at the health center, the most common group-provider facility. And because the number of solo-provider facilities is much larger than the number of group-provider facilities the total number of patients seen by all the solo-provider facilities is potentially similar to, if not larger than, that seen by all the group-provider facilities. What is clear is that we need much better information about the contribution this group makes to outpatient care.

Indonesia's health information system concentrates mostly on obtaining information about the public sector. The results of this study indicate just how partial is the picture provided by the concentrating on the public sector in this way. Across the 15 districts studied here 90% of the health facilities are in the private sector and most are not systematically and regularly included in the health information system, either as facilities that provide health care and therefore are a part of the whole health system, or as facilities that see patients and should be included in disease reporting systems and interventions to improve overall service quality.

Overall, Indonesia's approach to the development of health services achieved a wide distribution of health facilities and staff. Although perhaps unintended by those who devised the Bandung Plan, the vast majority of the health facilities (86%) are solo-provider facilities in which the provider and the facility are synonymous. Most of the facilities in a district are at the sub-district level and below. Through the village midwife and nurses these solo facilities were distributed more widely than health centers and even sub-centers. At the same time, and continuing to this day, there are centripetal forces working to contract the distribution of facilities and providers. The concentration of doctors in urban areas was documented in the 1990s [8] and is only likely to have intensified since. Further, doctors assigned to health centers have always found opportunities to be away including, in some cases, spending most of their time at private practices in urban areas. District health officials report that midwives have tended to move to health centers from the villages and there are widespread anecdotes (but no quantitative evidence) from health sector administrators indicating that many village midwives spend little time in the village to which they have been assigned. Many, if not most, young graduates in all professional groups have a preference for urban areas for family and lifestyle reasons. The result of these preferences is a high rate of absenteeism from the public multi-provider facilities, particularly health centers, as well as the solo-provider facilities staffed by the village midwife – an independent multi-country survey found absenteeism rates of 40% in the health sector in Indonesia, the highest of all countries surveyed [17].

Nevertheless, for a period Indonesia was able to distribute facilities more evenly by locating health centers at the sub-district level and conscripting staff to work there. Conscription is no longer an option for the government. The challenge now is to determine the level of distribution of facilities and providers the government should aim for and identify the ways in which this can be achieved. No matter how much we would like it to be the case, it seems clear that the level of distribution achieved in the 1980s is not possible now – the centripetal forces are winning.

As the various levels of government consider the future direction of the health system the distribution of facilities is a critical question, and the role of the health center, central to the distribution achieved so far, in that new system is important. The question is how to re-define the health center (its role, staffing and financing) in such a way that the distribution of facilities and staff makes a more effective health system for the Indonesia of the 21^st ^century – an Indonesia in which there is no coercion of health staff, in which the road infrastructure, though still needing much improvement, is a great deal better than 40 years ago so that patient mobility is much improved, an Indonesia in which the population has higher levels of income, disease patterns are changing, and the consumer prefers different types of services than was the case in the 1970s and 1980s.

A new vision is needed for the health sector, a vision which addresses the questions of the types, roles and distribution of health facilities and providers needed to tackle the health problems of the next 50 years. There are important issues to be addressed in the health sector. Can the government rise to meet the challenge?

## Conclusion

The relatively good distribution of health facilities in Indonesia was achieved through establishing public hospitals at the district level and public health centers at the sub-district level, both staffed through a system of compulsory service for doctors, nurses and midwives. Subsequently, these public sector staff also established solo-provider facilities for their own private practice; these solo-provider facilities, of which nurses contribute almost half, comprise the largest category of outpatient care facilities, but most are not included in official statistics. Now that Indonesia no longer has mandatory service for newly graduated doctors, nurses and midwives, it will have difficulty maintaining the distribution of facilities and providers established through the 1980s. The current challenge is to envision a new health system that responds to the changing health needs of the population as well as to the changes in distribution of health facilities.

## Competing interests

The authors declare that they have no competing interests.

## Authors' contributions

PH conceived the study, analyzed results and drafted the manuscript. NPH provided input on study design, supervised data collection in West Java Province, assisted with interpretation of results, and reviewed manuscript.

## Appendix 1. Endnotes

^1 ^Since 1984, the MEASURE DHS (Demographic and Health Surveys) project has provided technical assistance to more than 200 surveys in 75 countries, advancing global understanding of health and population trends in developing countries. DHS has earned a worldwide reputation for collecting and disseminating accurate, nationally representative data on fertility, family planning, and maternal and child health as well as child survival, HIV/AIDS, malaria, and nutrition. The MEASURE DHS project is funded by USAID with contributions from other donors. The project is implemented by ORC Macro, which since October 2003 has been partnering with four internationally experienced organizations to expand access to and use of the DHS data:

• The Johns Hopkins Bloomberg School of Public Health/Center for Communication Programs

• PATH

• Casals and Associates

• Jorge Scientific Corporation (JSC).

(see )

^2 ^A health facility is defined as a physical structure from which one or more health qualified providers (doctor, nurse, midwife) offer health services, both preventive and curative.

^3 ^A health service is defined as private if a charge is made for the service and the provider retains the money paid for the service whether or not the provider also receives a salary or wage from the public purse. A facility from which a single health care provider offers health care services is defined as a solo-provider facility.

## References

[B1] World Bank (2006). Making the New Indonesia Work for the Poor. Indonesia Poverty Assessment Report.

[B2] World Bank (2007). Spending for development: making the most of Indoinesia's new opportunities. Indonesia Public Expenditure Review.

[B3] Ron Duncan, McLeod Ross H, McLeod Ross H, MacIntyre Andrew (2007). The state and the market in democratic Indonesia. Indonesia: democracy and the promise of good governance.

[B4] Leimena J (1956). Public health in Indonesia Problems and planning.

[B5] Departemen Kesehatan (1975). Pelaksanaan Program Pembangunan Bidang Kesehatan Tahun Ke-Lima Pelita I (1973/1974).

[B6] Departemen Kesehatan (1997). Rencana Pembangunan Lima Tahum Ketujuh Bidang Kesehatan, 1998/1999 – 2003/2004.

[B7] Achmad J (1999). Hollow development: the politics of health in Soeharto's Indonesia.

[B8] World Bank (1994). Indonesia's Health Work Force: issues and options World Bank Report No 12835-IND, 12835-IND.

[B9] Heywood P, Harahap NP (2009). Human resources for health at the district level in Indonesia: the smoke and mirrors of decentralization. Human Resources for Health.

[B10] World Bank (2003). Decentralizing Indonesia: a regional public expenditure review – overview report.

[B11] Heywood P, Harahap NP (2009). Public funding at the district level in Indonesia – sources, flows and contradictions. Health Res Policy Syst.

[B12] World Bank (1997). Safe Motherhood Project: a partnership and family approach. Project Appraisal Document.

[B13] BPS-Statistics Indonesia (2003). Indonesia Demographic and Health Survey 2002–2003.

[B14] World Bank (2007). Spending for development: making the most of Indonesia's new opportunities Indonesia Public Expenditure Review.

[B15] Sciortino R (1995). Care-takers of cure An anthropological study of health centre nurses in rural Central Java.

[B16] Barber SL, Gertler PJ, Harimurti P (2007). Differences in access to high-quality outpatient care in Indonesia. Health Affairs.

[B17] Chaudhury N, Hammer J, Kremer M, Muralidharan K, Rogers FH (2006). Missing in action: teacher and health worker absence in developing countries. Journal of Economic Perspectives.

